# A New Method for Screening Thalassemia Patients Using Mid-Infrared Spectroscopy

**DOI:** 10.3390/diagnostics16010067

**Published:** 2025-12-24

**Authors:** Kanjana Thumanu, Tanaporn Khamgasem, Somsamorn Sukpong, Rungrueang Phatthanakun, Rawiwan Puangplruk, Waraporn Tanthanuch, Buabarn Kuaprasert, Sukanya Tastub, Roengrut Rujanakraikarn, Saitip Tun, Tassanee Saovana, Thongperm Munkongdee, Sujintana Wongthong

**Affiliations:** 1 Synchrotron Light Research Institute (Public Organization), 111 University Avenue, Mueang District, Nakhon Ratchasima 30000, Thailand; khemkasam@hotmail.com (T.K.); rungrueang@slri.or.th (R.P.); waraporn@slri.or.th (W.T.); buabarn@slri.or.th (B.K.); sukanya@slri.or.th (S.T.); roengrut@slri.or.th (R.R.); 2 Blood Testing Section, Regional Blood Centre 5th, The Thai Red Cross Society, Nakhon Ratchasima 30000, Thailand; somsamorn.s@redcross.or.th; 3 Regional Health Promotion Center 9 Nakhon Ratchasima, 177 Moo 6, Khok Kruat Subdistrict, Mueang District, Nakhon Ratchasima 30000, Thailand; rawiwann.puangplrukk.68@gmail.com (R.P.); saitip_t@yahoo.co.uk (S.T.); 4 Medical Technology, Faculty of Allied Health Sciences, Nakhon Ratchasima College, 290 Moo 2, Mittraphap Road, Ban Mai, Mueang District, Nakhon Ratchasima 30000, Thailand; tassanee@nmc.ac.th; 5 Thalassemia Research Center, Institute of Molecular Biosciences, Mahidol University, Nakhon Pathom 73170, Thailand; thongperm.mun@mahidol.ac.th; 6 Faculty of Public Health, Mahasarakham University, Khamriang Subdistrict, Kantarawichai District, Mahasarakham 44150, Thailand

**Keywords:** thalassemia, infrared microspectroscopy, blood samples

## Abstract

**Background/Objectives**: Thalassemia is an inherited blood disorder caused by defects in hemoglobin production, where an imbalance or reduction in globin-chain synthesis impairs normal red cell development and results in anemia of varying severity. The disease is classified into α-thalassemia and β-thalassemia according to the affected globin genes. In recent years, infrared (IR) Microspectroscopy has gained increasing attention for blood analysis because it is rapid, label-free, and capable of detecting subtle biochemical alterations. **Method**: In this study, we analyzed hemoglobin lysate collected from clinically characterized normal, carriers, and thalassemia patients (*n* = 333) using IR Microspectroscopy combined with multivariate statistical methods, including Principal Component Analysis (PCA) and Partial Least Squares Discriminant Analysis (PLS-DA). This approach enabled us to investigate how spectral features correspond to disease status across a range of genotypes commonly encountered in clinical practice. **Results**: Clear intergroup spectral differences were observed, and the classification models demonstrated diagnostic performance with sensitivity and specificity of approximately 80–90%. Because the technique is non-destructive, requires no chemical reagents, and allows direct biochemical profiling of hemoglobin, it offers practical advantages over conventional hematologic or molecular assays. **Conclusions**: These findings support the potential of IR-based spectral analysis as a complementary tool for thalassemia screening. Looking ahead, incorporating advanced machine learning algorithms with IR Microspectroscopy may further enhance early detection, improve risk stratification, and strengthen prevention and management strategies in routine clinical workflows.

## 1. Introduction

Thalassemia is an inherited hematologic disorder characterized by impaired synthesis of globin chains, the protein components of hemoglobin responsible for oxygen transport throughout the body. The disease follows an autosomal recessive inheritance pattern and is highly prevalent in Thailand. Clinically, thalassemia can be categorized into two principal groups: the Disease group, comprising affected patients, and the Carrier group, consisting of individuals who harbor thalassemia mutations without manifesting symptoms. These groups display varying degrees of severity and distinct clinical characteristics. Severe forms may lead to fetal hydrops and perinatal death, whereas moderate and mild cases commonly present with mild anemia and hepatosplenomegaly, which may worsen during febrile episodes. Although asymptomatic, individuals in the Carrier group can transmit the mutated gene to their offspring [1,2,3,4,5].

In Thailand, the combined prevalence of the Disease and Carrier groups is estimated to reach up to 40% of the screened population, posing a significant public health challenge. Current screening programs primarily target married couples; however, the diagnostic workflow remains costly and labor-intensive. The development of more accessible, rapid, and cost-effective screening methods is therefore essential to strengthen national healthcare capacity [6,7,8,9].

The aim of this study is to establish a practical and cost-efficient diagnostic strategy for thalassemia screening by identifying and classifying biochemical alterations in hemoglobin lysate samples from normal, carrier, and disease + symptom groups using infrared (IR) Microspectroscopy. This vibrational spectroscopic technique generates a distinctive molecular fingerprint that reflects the chemical composition and structural organization of biomolecules such as proteins, lipids, and nucleic acids [10,11]. IR Microspectroscopy offers multiple advantages: it is non-destructive, label-free, inexpensive, rapid, and requires minimal sample preparation without chemical reagents [12,13,14,15,16,17,18,19,20,21,22,23,24,25,26,27]. The acquired spectral data will be analyzed using artificial intelligence (AI)and multivariate statistical techniques to develop predictive classification models and an IR spectral database capable of accurately differentiating thalassemia types [6,28,29,30]. Thalassemia comprises several clinically relevant subtypes including β-thalassemia (β^0^ and β^+^), α-thalassemia (α^0^ and α^+^), hemoglobin H disease (HbH), and hemoglobin E (HbE) and reliable classification is crucial for genetic counseling, family planning, and clinical management, especially among carrier couples [31,32,33,34].

In this study, IR Microspectroscopy combined with Principal Component Analysis (PCA) and Partial Least Squares Discriminant Analysis (PLS-DA) will be used to establish a comprehensive spectral database and construct mathematical models capable of distinguishing biochemical severity between carrier and disease + symptom groups. Model performance will be assessed through sensitivity, specificity, and predictive accuracy, providing a solid foundation for integrating spectroscopic-based diagnostics into clinical thalassemia screening programs.

## 2. Materials and Methods

**Figure 1:**
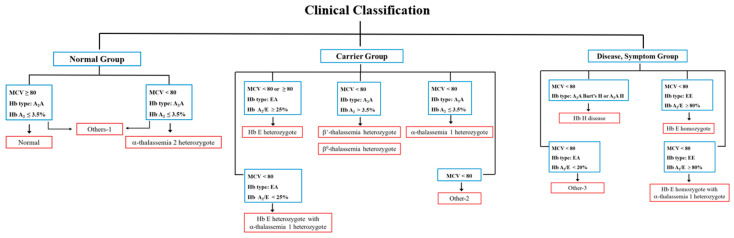
Clinical Criteria for Genotypic and Phenotypic Classification of Thalassemia Study Groups.

The study participants were categorized into three primary groups—Normal, Carrier, and Disease + Symptom—based on integrated hematological and genotypic assessments. Each primary group was subsequently divided into more specific genotypic or phenotypic subgroups, determined by molecular characteristics verified through hemoglobin typing and/or DNA analysis. A comprehensive overview of all subgroup classifications is provided in Table 1, along with the corresponding clinical classification illustrated in Figure 1, Table 2 and Supplementary Tables S1–S12.

### 2.1. Analysis of Blood Samples Using IR Microspectroscopy

Blood samples were analyzed using a Fourier Transform Infrared (FTIR) spectrometer (Tensor 27, Bruker Optics, Ettlingen, Germany) coupled with an IR Microscope (Hyperion 3000, Bruker Optics) equipped with an MCT detector. Spectral acquisition and instrument control were performed using OPUS 7.5 software (Bruker Optics, Germany). Measurements were conducted in transmission mode using a 15X objective lens (Bruker Optics, Ettlingen, Germany,) with a spectral resolution of 4 cm^−1^ and a background scan of 64 scans. The spectral range was recorded between 4000 and 600 cm^−1^, and each group of samples was prepared by placing three drops of the sample on the IR window for three replications, resulting in a total of about 45 spectra/sample to represent each patient. After that, spectra were analyzed to remove outliers within the groups by performing the same data preprocessing protocol using the Unscrambler 10.1

### 2.2. Spectral Preprocessing and Multivariate Data Analysis

All spectra were subjected to data preprocessing prior to analysis. The data preprocessing was conducted using baseline corrections and then normalized using EMSC within the spectral regions from 3171 to 2788 cm^−1^ and from 1750 to 800 cm^−1^. The preprocessed spectra were then analyzed using Principal Component Analysis (PCA) to identify and compare biochemical variations among different types of thalassemia. The preprocessing and PCA were performed using The Unscrambler X version 10.3 (CAMO Software, Oslo, Norway).

### 2.3. Construction of a Spectral Database for Thalassemia Classification

The processed data were compiled into a database to classify the IR spectral characteristics of different thalassemia subtypes, including Normal, Carrier and Disease + Symptom groups. Further classification and predictive modeling were carried out using Partial Least Squares Discriminant Analysis (PLS-DA) with The Unscrambler X version 10.3 (CAMO Software, Norway). Samples were categorized based on disease severity into Disease + Symptom, Carrier, and Normal groups, with at least 30 samples per phenotype. PLS-DA was then employed to analyze and validate the classification performance and model discrimination capability among these groups [35,36,37,38,39,40,41,42,43,44,45,46,47].

**Table 2:** Clinical and Hematological Criteria for Thalassemia Classification.

Parameter	Criterion (Screening/Diagnostic Cut-Off)	References
RBC count (×10^6^/µL)	>5.0 indicates microcytic erythrocytosis (suggestive of thalassemia trait)	[48,49]
Hemoglobin (Hb, g/dL)	<12.0 indicates anemia; <10.0 suggests moderate to severe anemia	[48,50]
Hematocrit (Hct, %)	<36% considered anemic threshold	[48,49]
Mean Corpuscular Volume (MCV, fL)	<80 fL indicates microcytosis	[48,51,52]
Mean Corpuscular Hemoglobin (MCH, pg)	<27 pg indicates hypochromia	[48,50]
Red Cell Distribution Width (RDW, %)	>14% indicates anisocytosis; may support thalassemia or IDA	[49,51]
HbA_2_ (%)	>3.5% diagnostic for β-thalassemia trait	[50,51]
HbE (%)	25–30% diagnostic for HbE trait	[50,51]
HbF (%)	>1% suggests β-thalassemia intermedia or major	[49,51]

## 3. Results and Discussion

Within the broader “Normal” category, three distinct clusters were observed: true normal samples (Group 10), α-thalassemia 2 heterozygotes (Group 22), and individuals carrying Hb Constant Spring or Hb Paksae’ heterozygotes (Group 25). Groups 10 and 22 cluster closely on the dendrogram, reflecting their minimal differences in globin synthesis and overall hemoglobin composition; as a result, their IR spectra remain largely similar, particularly in the Amide I and II regions. In contrast, Group 25 forms a clearly separated branch, consistent with the structural instability and altered folding caused by non-deletional α-globin variants. These molecular effects produce more pronounced spectral deviations, explaining why their IR profiles diverge from the other two subgroups.

### 3.1. IR Microspectroscopy and Functional Group Analysis

**Figure 2:**
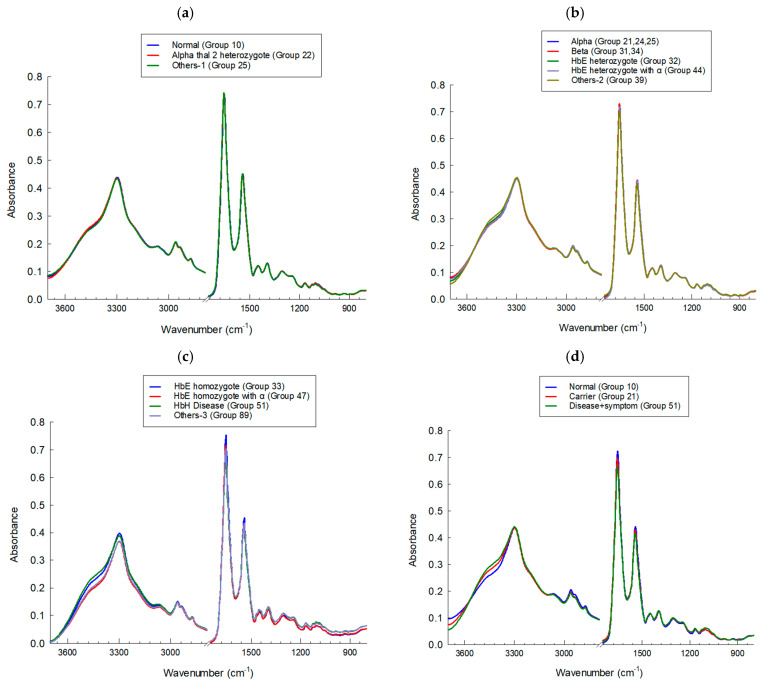
Average FTIR spectra of Hb lysate red blood samples from the (**a**) Normal group (*n* = 100), (**b**) Carrier group (*n* = 145), (**c**) Disease + Symptom group (*n* = 88) and (**d**) Comparison of the 3 groups (*n* = 93) recorded in the 4000–900 cm^−1^ range using IR microscopy (100 × 100 µm, 4 cm^−1^ resolution, 64 scans). Spectra were preprocessed using baseline correction, smoothing, and Extended Multiplicative Signal Correction (EMSC), followed by PCA.

FTIR spectra of hemoglobin lysate were collected across all subgroups, with roughly 45 spectra recorded per sample. After removing obvious outliers, the data were explored using Principal Component Analysis (PCA) and Figure 2 presents the average FTIR spectra of hemoglobin (Hb) lysate red blood cell samples. The dataset itself covered a fairly wide range of clinically encountered thalassemia genotypes, such as the true Normal group (Group 10), α-thalassemia 2 heterozygote (Group 22), Hb Constant Spring or Hb Paksae’ heterozygotes (Group 25), other α-thalassemia genotypes (Groups 21, 24), several types of β-thalassemia heterozygote (Groups 31, 34), Hb_E heterozygotes (Group 32), compound heterozygotes such as Hb_E with α-thalassemia 1 heterozygote (Group 44), Hb_E homozygotes with α-thalassemia 1 heterozygote (Group 47), Hb_E homozygotes (Group 33), and additional less common categories (Groups 39, 51, 89). Most groups contained between 15 and 30 samples. For comparison in the PCA plots, three groups were selected to represent the broader clinical categories: Disease + Symptom (Group 51), Carrier (Group 21), and Normal (Group 10).

Across the dataset, the FTIR spectra showed the expected absorption bands associated with major biochemical components of hemoglobin (Table 3). Clear protein-related features were present, including Amide A and B, as well as CH_2_ and CH_3_ stretching signals linked to protein side chains. The Amide I region (around 1600–1700 cm^−1^) captured variations in secondary structure content, while the Amide II region (1500–1600 cm^−1^) reflected peptide-bond vibrations. Some differences between the Disease + Symptom and Carrier groups could also be seen in the mid-spectral region, particularly near 1450 and 1350 cm^−1^, where side-chain deformation modes appear.

Taken together, the spectra displayed consistent signatures from both the peptide backbone and the side-chain environments of hemoglobin. The variation observed among groups reflects real biochemical diversity and provides a reasonable basis for distinguishing thalassemia categories in relation to disease severity.

### 3.2. Cluster Analysis of FTIR Spectra of Hb Lysate

**Figure 3:**
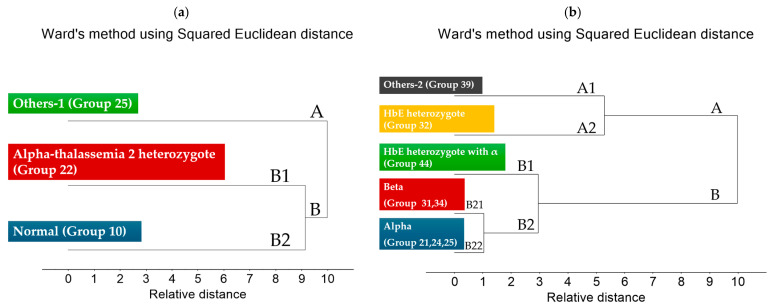

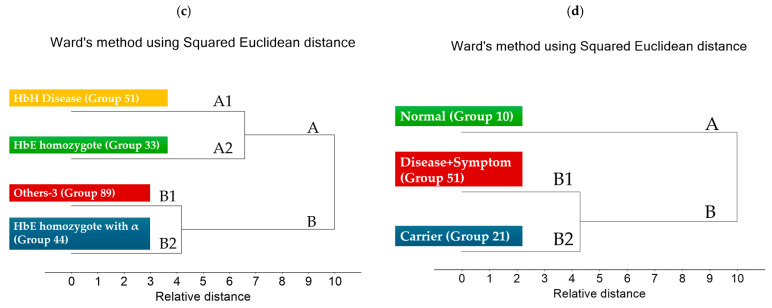
Dendrogram generated from Unsupervised Hierarchical Cluster Analysis (UHCA) of FTIR spectra from the (**a**) Normal (*n* = 100), (**b**) Carrier (*n* = 145), (**c**) Disease + Symptom (*n* = 88) and (**d**) Comparison of the 3 groups (*n* = 93) using Ward’s algorithm, covering spectral regions 781–1751 cm^−1^ and 2788–3712 cm^−1^.

The dendrogram (Figure 3a) shows that Others-1 (Group 25) forms a clearly separate branch, indicating strong spectral and biochemical differences due to its non-deletional α-globin variants. In contrast, Alpha-thalassemia 2 heterozygotes (Group 22) cluster closely with the Normal group (Group 10), reflecting their similar hemoglobin composition and minimal structural alterations. Overall, Group 25 is distinctly separated, while Groups 22 and 10 show high similarity and form a joint cluster.

The dendrogram (Figure 3b) separates the subgroups into two major clusters. Others-2 (Group 39) and Hb_E heterozygotes (Group 32, A2) form Cluster A because Hb_E is a structural hemoglobin variant, producing characteristic changes in the IR spectra that differ from simple reductions in globin synthesis. Cluster B contains the α and β thalassemia subtypes, where the primary defect is reduced globin-chain production, leading to a different biochemical and spectral pattern. Within Cluster B, HbE heterozygotes with α-thalassemia (Group 44, B1) show an intermediate profile, as their spectra reflect the combined effects of both a structural HbE variant and α-globin deficiency.

The dendrogram (Figure 2c) divides the subgroups into two main clusters. Cluster A includes Hb_H disease (Group 51) and Hb_E homozygotes (Group 33), which cluster together because both conditions cause major imbalances in globin-chain production, leading to similar biochemical and spectral profiles. Cluster B consists of Others-3 (Group 89) and Hb_E homozygotes with α-thalassemia heterozygotes (Group 44); these groups separate from Cluster A due to their more complex or compound genetic defects, which generate distinct IR spectral patterns. Overall, the clustering reflects the underlying molecular differences between severe globin-chain imbalance (Cluster A) and mixed or atypical mutations (Cluster B).

When integrating all datasets (Figure 3d), two major clusters were observed: Cluster A (Normal) and Cluster B (Carrier + Disease). Cluster B could be further divided into B1 (Carrier) and B2 (Disease + Symptom), clearly demonstrating the ability of FTIR spectral profiles to distinguish between severity levels of thalassemia. When correlated with FTIR spectral features, these group separations were associated with specific functional group variations, particularly within the Amide I (1600–1700 cm^−1^) and Amide II (1500–1600 cm^−1^) regions related to protein secondary structure. These bands played a key role in differentiating Normal samples from Carrier and Disease + Symptom groups. In addition, signals corresponding to side-chain vibrations (CH_2_, CH_3_) and C–O stretching modes (1400–1000 cm^−1^) contributed to the discrimination of Disease + Symptom subtypes with greater specificity.

Overall, FTIR Microspectroscopy combined with hierarchical cluster analysis (HCA) proved effective for molecular-level screening and classification of thalassemia. The technique shows strong potential as a biomarker-based approach for predicting disease severity and supporting early diagnosis in clinical practice.

### 3.3. Principal Component Analysis (PCA) and Partial Least Squares Discriminant Analysis (PLS-DA) of FTIR Spectra of Hb Lysate

**Figure 4:**
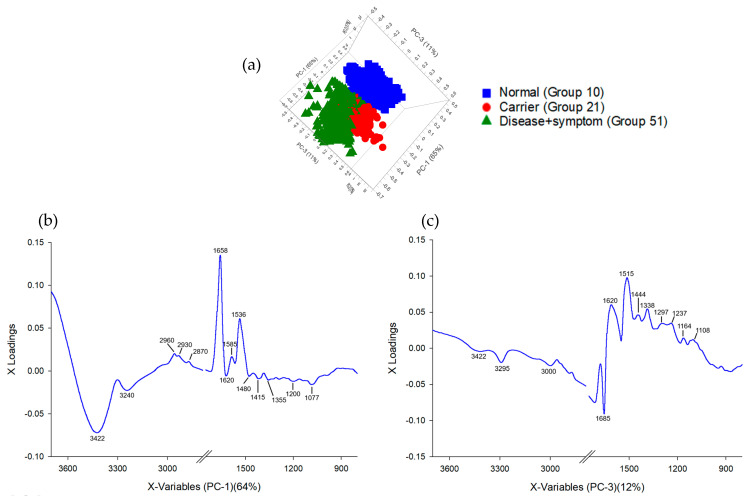
Principal Component Analysis (PCA) of Hb lysate samples classified by thalassemia severity. (**a**) PCA score plot of Normal (Group 10), Carrier (Alpha-thalassemia 1 heterozygotes, Group 21), and Disease + Symptom (Hb_H Disease, Group 51) groups (*n* = 31). (**b**) PCA loading plot for PC-1 (64% explained variance) of FTIR spectra. (**c**) PCA loading plot for PC-3 (12% explained variance) of FTIR spectra.

The PCA of the FTIR spectra from the hemoglobin lysate (Figure 4a) showed that most of the variation in the dataset could be explained by the first three components, which together captured roughly 76% of the total variance (PC-1: 64%, PC-3: 12%). When the scores were plotted, the Normal samples tended to cluster separately from the Carrier and Disease + Symptom groups along PC-1. The second and third components added some additional separation, mainly helping distinguish the Carrier group from the Disease + Symptom group.

From the loading patterns, PC-1 (Figure 4b) mainly reflected differences in several broad spectral regions, including the Amide A zone, CH_2_/CH_3_ stretching, and the Amide I–II regions. These areas often relate to variations in secondary-structure features of hemoglobin, which explains why PC-1 was effective at separating normal from thalassemic profiles. PC-3 (Figure 4c) included features within the Amide I and II regions as well, along with peaks in the lower wavenumber region linked to Amide III and C–O stretching, which tend to reflect more detailed changes in secondary structure or side-chain environments. Taken together, the PCA results suggest that the spectral differences associated with protein backbone structure and side-chain chemistry are sufficient to differentiate Normal, Carrier, and Disease + Symptom groups, even before applying a supervised method.

### 3.4. Correlation Loadings Analysis of PCA from FTIR Spectra of Hb Lysate

**Figure 5:**
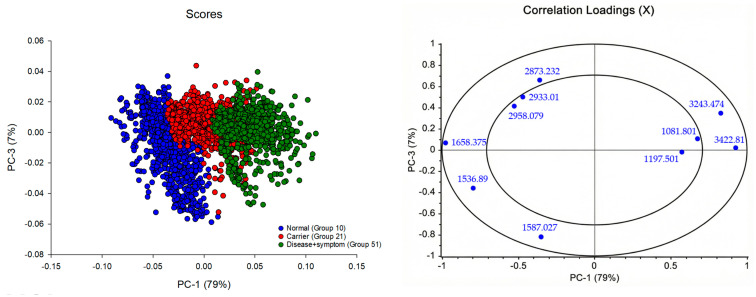
PCA score plot (PC-1 vs. PC-3) and correlation loading plot of FTIR spectra of Hb lysate samples, demonstrating clear separation among Normal, Carrier, and Disease + Symptom groups.

**Figure 6:**
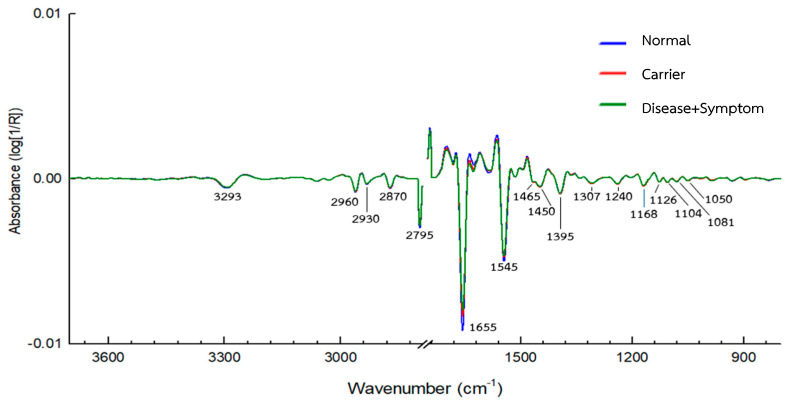
Second-derivative FTIR spectra of Hb lysate samples from Normal, Carrier, and Disease + Symptom groups, highlighting the characteristic absorption bands associated with specific molecular vibrations.

The PCA results showed that most of the variation across the FTIR spectra was captured by PC-1, which alone explained about 79% of the total variance. This component largely drove the separation between the Normal samples and those from the Carrier and Disease + Symptom groups, which matches what was seen in the score plot (Figure 5 and Figure 6). When looking at the loadings, the more positive contributions on PC-1 were mainly from the higher-wavenumber regions around 3243 and 3422 cm^−1^ and from the CH_2_/CH_3_ stretching range between 2873 and 2958 cm^−1^. These patterns generally relate to differences in hydrogen bonding and the chemical environment of protein side chains, which appear to shift noticeably in thalassemic samples compared with normal samples. On the opposite side, the signal at 1658 cm^−1^ (Amide I) loaded strongly in the negative direction and was more characteristic of the Normal group, suggesting a more stable arrangement of secondary-structure elements in these samples. Peaks commonly linked to Amide II, such as those near 1536 and 1587 cm^−1^, were more associated with the Carrier and Disease + Symptom samples, which is consistent with the altered peptide-bond vibrations expected from structural changes in thalassemic hemoglobin.

PC-3, although accounting for only 7% of the variance, still contributed to distinguishing the Carrier group from the Disease + Symptom group. The loadings at approximately 1081 and 1187 cm^−1^, typically linked to Amide III or C–O stretching and certain glycoprotein-related motions, indicated shifts in side-chain behaviour and other secondary-structure features that differ between these two categories.

Overall, the combined loading patterns point to changes in both peptide-backbone vibrations and the chemical environments of protein side chains. These spectral differences are in line with the underlying biochemical alterations seen in varying severities of thalassemia and help explain why the three groups can be separated effectively in PCA.

**Figure 7:**
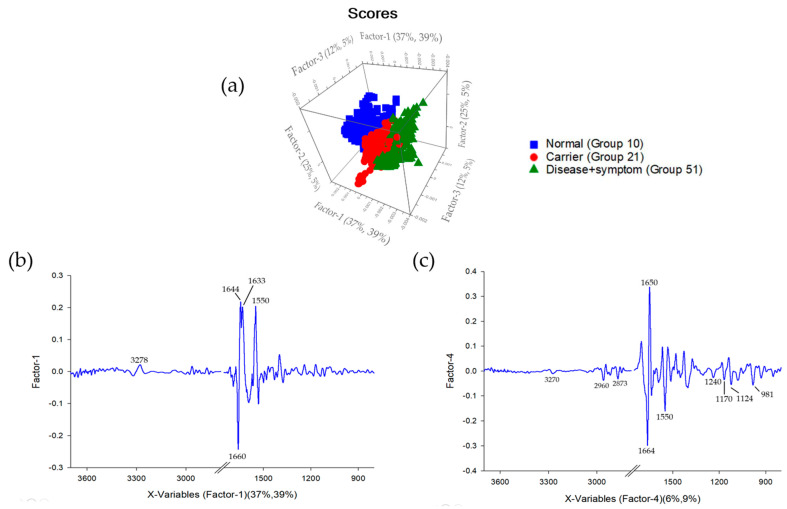
(**a**) Three-dimensional PLS-DA score plot of Factor-1 and Factor-4, showing clear separation among Normal (Group 10), Carrier (Group 21), and Disease + Symptom (Group 51). (**b**) Factor-1 loading plot (37–39% variance), highlighting major spectral differences between Normal and other groups. (**c**) Factor-4 loading plot (6–9% variance), showing subtle variations that refine separation between Carrier and Disease + Symptom.

Factor-1 and Factor-4 provide a comprehensive molecular fingerprint of thalassemia severity. Factor-1 (Figure 7b) reflects primary disruptions of the protein backbone; the loading plot for Factor-1 highlights significant contributions at Amide I 1660 cm^−1^, as well as Amide II at 1587 and 1533 cm^−1^ [56]. Factor-4 (Figure 7c) captures secondary structural perturbations, including glycoprotein and side-chain alterations. Factor-4 provides finer resolution between groups, particularly between the Carrier and Disease + Symptom categories. The positive displacement in the score plot aligns with inverse features observed in the loading plot for Factor-4, highlighting significant contributions in the higher-wavenumber regions around 3270 cm^−1^, from the CH_2_/CH_3_ stretching range between 2873 and 2960 cm^−1^, the Amide III region (1240 cm^−1^),C–O stretching and glycoprotein-related regions (1170 and 1124 cm^−1^), as well as at 981 cm^−1^, a marker associated with carbohydrate or aromatic side-chain vibrations [57]. Elevated intensities in these regions in Disease + Symptom samples indicate perturbations in secondary structure stability, modifications of glycoprotein-associated residues, and changes in aromatic amino-acid environments. Together, this integrated interpretation demonstrates the effectiveness of FTIR-based PLS-DA in resolving both major and subtle biochemical differences among Normal, Carrier, and Disease + Symptom groups, supporting its potential as a diagnostic tool for thalassemia classification [59,61].

These findings underscore the sensitivity of FTIR spectroscopy in capturing nuanced conformational changes within hemoglobin molecules, which are often imperceptible through conventional hematological assays. By integrating multivariate PLS-DA analysis, subtle variations in protein secondary structure, glycoprotein modifications, and side-chain microenvironments can be quantitatively distinguished across phenotypic groups. The approach not only facilitates accurate classification of thalassemia subtypes but also provides mechanistic insights into the molecular underpinnings of disease progression. Consequently, this methodology holds promise for enhancing diagnostic precision, guiding therapeutic decisions, and informing future research into structure-function relationships in hemoglobinopathies.

**Figure 8:**
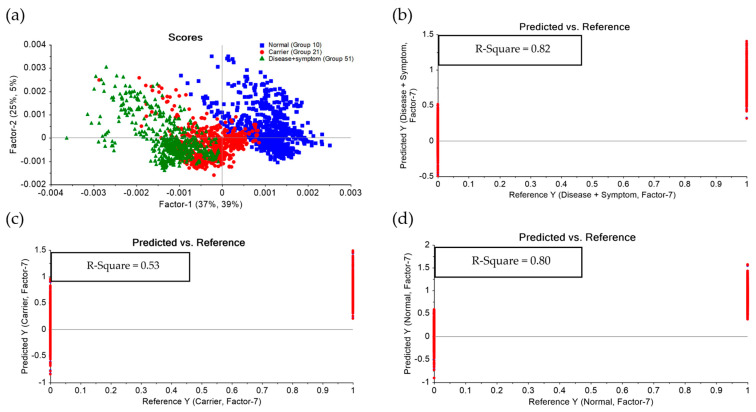
(**a**) PLS-DA analysis of Hb lysate samples classified into three severity levels: Normal, Carrier, and Disease + Symptom. (**b**–**d**) Calibration curves for Disease + Symptom, Carrier, and Normal groups, respectively. Each group contained 31 samples (Normal: Normal; Carrier: Alpha-thalassemia 1 heterozygotes (Group 21); Disease + Symptom: Hb_H Disease (Group 51), with a total of 3021 spectra analyzed).

**Figure 9:**
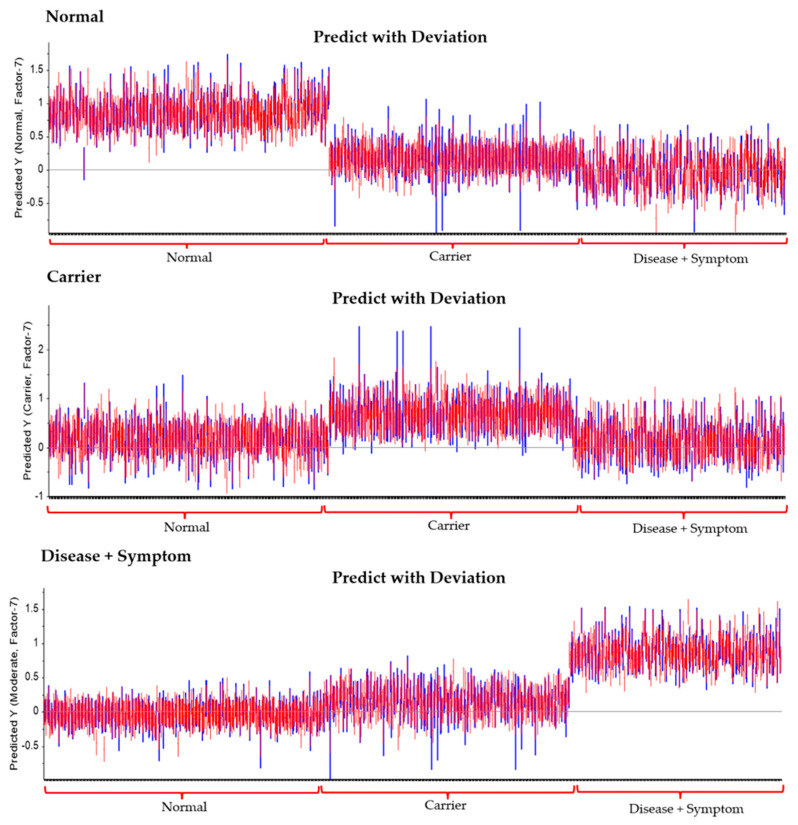
PLS-DA prediction model for Hb lysate samples, demonstrating classification among Normal (Normal, Group 10), Carrier (Alpha-thalassemia 1 heterozygotes, Group 21) and Disease + Symptom (Hb_H Disease, Group 51) groups. Each group contained 31 samples (*n* = 31), with a total of 3021 spectra analyzed.

Based on the experimental results, further analysis was conducted using Partial Least Squares Discriminant Analysis (PLS-DA) to classify the severity levels among the Disease + Symptom, Carrier, and Normal groups. For the classification model, the Normal group was represented by samples of Normal (Group 10), the Carrier group by Alpha-thalassemia 1 heterozygotes (Group 21), and the Disease + Symptom group by Hb_H Disease (Group 51) samples. The model performance was evaluated in terms of the R^2^ coefficient, where the Disease + Symptom group achieved an R^2^ of 0.82, the Carrier group achieved R^2^ = 0.80, and the Normal group achieved R^2^ = 0.53, as shown in Figure 8 and The PLS-DA model demonstrated high diagnostic accuracy across all categories. Specifically, it achieved 98% sensitivity and 99% specificity for the Normal group, and 99% sensitivity and 100% specificity for the Disease + Symptom group. The Carrier group was classified with a sensitivity of 81% and a specificity of 91% shown in Figure 9 and Table 4.

**Figure 10:**
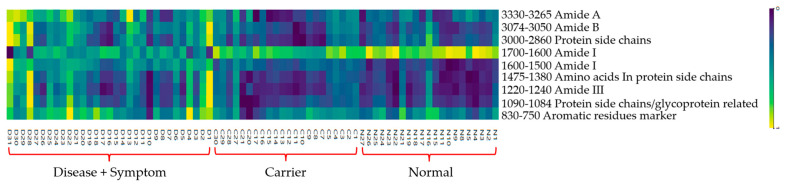
Heatmap of FTIR spectra of Hb lysate samples comparing absorbance values across functional group regions among Normal (N), Carrier (C), and Disease + Symptom (D) groups. The analysis was conducted using Orange (Quasar) with normalization and Min–Max scaling applied per functional group.

The analysis showed that, as illustrated in the heatmap (Figure 10), The Normal group exhibited the highest absorbance intensity in the Amide I region (1600–1700 cm^−1^), particularly near ~1650 cm^−1^, reflecting a well-preserved α-helix–rich secondary structure in hemoglobin. Higher absorbance was also observed in the Amide II region (1500–1600 cm^−1^) and in amino-acid side-chain bands at 1452 and 1387 cm^−1^, indicating stable peptide-bond vibrations and intact side-chain environments typical of healthy blood samples.

The Carrier group displayed moderately reduced absorbance in the Amide I and Amide II regions relative to the Normal group, yet remained higher than the Disease + Symptom group across much of the spectrum. These patterns suggest partial but not severe alterations in protein structure associated with carrier status. Slightly lower absorbance in the protein side-chain regions (3000 and 2875 cm^−1^) further indicates mild perturbations to lateral-chain environments.

In contrast, the Disease + Symptom group exhibited the most pronounced deviations. Absorbance in both Amide I and Amide II decreased substantially compared with the Normal and Carrier groups, signaling a loss of secondary-structure stability. Conversely, this group showed the highest absorbance in the Amide A region (3265–3329 cm^−1^, N–H stretching), consistent with hydrogen-bond disruption and potential protein unfolding or aggregation. Elevated signals in the Amide III region (1240–1220 cm^−1^) and in aromatic-residue markers (830–750 cm^−1^) further indicate secondary-structure alterations and changes involving aromatic amino acids such as tyrosine and phenylalanine [56].

In summary, the Normal group demonstrated strong absorbance in spectral features associated with stable protein backbone and side-chain structures (Amide I, Amide II, and amino-acid side chains). The Disease + Symptom group showed elevated absorbance in regions indicative of structural destabilization (Amide A, Amide III, and aromatic-residue bands), while the Carrier group exhibited intermediate characteristics, consistent with its mild and subclinical phenotype.

## 4. Conclusions

This study utilized infrared microspectroscopy to differentiate various forms of thalassemia and to develop a spectral database capable of identifying blood samples at risk of thalassemia. By establishing a comprehensive IR spectral library from blood samples categorized according to disease severity—Normal, Carrier, and Disease + Symptom—and applying Principal Component Analysis (PCA) and Partial Least Squares Discriminant Analysis (PLS-DA), we successfully distinguished the groups based on their underlying biochemical signatures. The analysis revealed clear molecular differences, particularly in protein secondary-structure features (Amide I region) and amino-acid side-chain vibrations, demonstrating that structural alterations in hemoglobin closely correlate with thalassemia severity.

In terms of diagnostic performance, the classification models achieved sensitivity and specificity values in the range of 80–90%, confirming the reliability and practical potential of this approach. Future work will involve expanding the dataset through additional sample collection from each thalassemia subtype, with particular emphasis on PCR-based genotyping of α-thalassemia 1 heterozygotes and α-thalassemia 2 heterozygotes. This will improve the resolution of Normal individuals without latent α-thalassemia mutations and enhance model robustness through AI-assisted spectral analysis. A petty patent titled “Process for Preparation and Measurement of Blood Samples” (Application No. 2503002000) has also been officially registered.

The next phase of this research will focus on integrating the developed system into a pilot thalassemia screening program at the Thai Red Cross Society, with the aim of establishing a practical, rapid, and cost-effective diagnostic tool for clinical implementation.

## Figures and Tables

**Table 1:** Genotypic and Phenotypic Classification of Thalassemia Study Groups.

		Group	Phenotype
Normal	Normal	10	Normal
	Alpha-thal 2 hetero	22	Alpha-thalassemia 2 heterozygote
	Others-1	25	Hb Constant Spring or Hb Paksaé heterozygote
Carrier	Alpha	21	Alpha-thalassemia 1 heterozygote
		24	Compound Alpha-thalassemia 2 heterozygote
		45	Hb E heterozygote with Alpha-thal 2 heterozygote
	Beta	31	Beta (0)-thalassemia heterozygote
		34	Beta (+)-thalassemia heterozygote
	HbE hetero	32	Hb E heterozygote
	HbE hetero with Alpha	44	Hb E heterozygote with Alpha-thalassemia 1 heterozygote
	Others-2	39	Hb E heterozygote with Alpha-thalassemia 2 heterozygote
Disease	HbE homo	33	Hb E homozygote
	HbE homo with Alpha	47	Hb E homozygote with Alpha-thalassemia 1 heterozygote
	HbH	51	Hb H Disease
	Others-3	89	EA Bart’s Disease

**Table 3:** Band assignments of the chemical structure and composition of hemoglobin.

Wavenumber (cm^−1^)	Assignments	Molecular Vibration of Functional Group	References
3290	Amide A	N-H Stretching	[53]
3050	Amide B	N-H Stretching	[53]
3000	Protein side chains	Symmetric and asymmetric of CH_2_	[54]
2875	Protein side chains	stretching vibrations of CH_3_	[54]
1600–1700	Amide I	C=O Stretching	[55]
1500–1600	Amide II	N-H Bending	[55,56,57,58,59]
1452	Amino acids in the protein side chains	Bending vibrations of CH_2_: δ (CH_2_)	[60]
1387	Amino acids in the protein side chains	Bending vibrations of CH_3_: δ(CH_3_)	[60]
1240–1220	Amide III Protein secondary structure	C–N stretch + N–H bending	[56]
1084–1090	protein side chains/glycoprotein-related vibration	C–O stretching	[23,56,61]
830–750	Aromatic residues marker	Aromatic ring breathing (Tyr, Phe)	[56]

**Table 4:** Sensitivity and specificity values calculated from the PLS-DA prediction analysis for model performance evaluation.

Group	Statistics	No. Vigor	Sensitivity	Specificity
Normal	True positive	378	0.98	0.99
	False Positive	6		
Carrier	True positive	272	0.81	0.91
	False Positive	62		
Disease + Symptom	True positive	287	0.99	1.00
	False Positive	3		

## Data Availability

The datasets used and/or analyzed during the current study are available from the corresponding author upon reasonable request.

## Supplementary Materials

The following supporting information can be downloaded at: https://www.mdpi.com/article/10.3390/diagnostics16010067/s1, Table S1: Genotypic and Phenotypic Classification of Thalassemia of Normal Group: Normal (Group 10); Table S2: Genotypic and Phenotypic Classification of Thalassemia of Normal Group: Alpha thalassemia 2 heterozygote (Group 22); Table S3: Genotypic and Phenotypic Classification of Thalassemia of Normal Group: Others-1 (Group 25); Table S4: Genotypic and Phenotypic Classification of Thalassemia of Carrier Group: Alpha (Group 21, 24 and 45); Table S5: Genotypic and Phenotypic Classification of Thalassemia of Carrier Group: Beta (Group 31 and 34); Table S6: Genotypic and Phenotypic Classification of Thalassemia of Carrier Group: HbE Heterozygote (Group 32); Table S7: Genotypic and Phenotypic Classification of Thalassemia of Carrier Group: Hb E heterozygote with Alpha-thalassemia 1 heterozygote (Group 44); Table S8: Genotypic and Phenotypic Classification of Thalassemia of Carrier Group: Others-2 (Group 39); Table S9: Genotypic and Phenotypic Classification of Thalassemia of Disease + Symptom Group: Hb E homozygote (Group 33); Table S10: Genotypic and Phenotypic Classification of Thalassemia of Disease + Symptom Group: Hb E homozygote with Alpha-thalassemia 1 heterozygote (Group 47); Table S11: Genotypic and Phenotypic Classification of Thalassemia of Disease + Symptom Group: Hb H disease (Group 51); Table S12: Genotypic and Phenotypic Classification of Thalassemia of Disease + Symptom Group: Others-3 (Group 89).
