# Supplementary material for: A New Method for Screening Thalassemia Patients Using Mid-Infrared Spectroscopy
Source: Diagnostics (Basel). 2025 Dec 24;16(1):67. doi: 10.3390/diagnostics16010067 (PMC12786254; doi:10.3390/diagnostics16010067)
Supplement: Supplementary file 1 [file diagnostics-16-00067-s001.zip › diagnostics-3986855-supplementary.pdf]

# Genotypic and Phenotypic Classification of Thalassemia Study Groups

**Table S1.** Genotypic and Phenotypic Classification of Thalassemia of Normal Group: Normal (Group 10)

| No. | RBC | Hb (g/dl) | Hct (%) | MCV (fl) | MCH (pg) | RDW (%) | Hb Typing | HbA2/HbE (%) | HbF (%) | PCR for $\alpha$-Thalassemia 2 3.7/4.2 kb deletion, CS/PS gene |
|---|---|---|---|---|---|---|---|---|---|---|
| **1** | 4.00 | 13.4 | 39.3 | 92.2 | 31.4 | 12.7 | $A_2A$ | 3.4 | 0.3 | Negative for $\alpha$-Thalassemia 2 and CS/PS gene |
| **2** | 5.48 | 14.8 | 45.6 | 83.2 | 27.0 | 13.7 | $A_2A$ | 2.9 | 0.9 | Negative for $\alpha$-Thalassemia 2 and CS/PS gene |
| **3** | 4.34 | 13.2 | 39.6 | 91.0 | 30.3 | 12.2 | $A_2A$ | 2.9 | 0.3 | Negative for $\alpha$-Thalassemia 2 and CS/PS gene |
| **4** | 4.72 | 13.0 | 39.7 | 84.2 | 27.6 | 13.5 | $A_2A$ | 3.0 | 0.5 | Negative for $\alpha$-Thalassemia 2 and CS/PS gene |
| **5** | 4.93 | 14.7 | 44.5 | 90.2 | 29.8 | 12.2 | $A_2A$ | 2.9 | 0.3 | Negative for $\alpha$-Thalassemia 2 and CS/PS gene |
| **6** | 5.84 | 15.0 | 46.5 | 49.6 | 25.7 | 15.6 | $A_2A$ | 3.0 | 0.5 | Negative for $\alpha$-Thalassemia 2 and CS/PS gene |
| **7** | 4.46 | 13.1 | 40.7 | 91.2 | 29.5 | 12.1 | $A_2A$ | 2.5 | - | Negative for $\alpha$-Thalassemia 2 and CS/PS gene |
| **8** | 4.38 | 13.1 | 39.3 | 89.8 | 29.9 | 13.2 | $A_2A$ | 3.3 | 0.2 | Negative for $\alpha$-Thalassemia 2 and CS/PS gene |
| **9** | 4.83 | 14.3 | 44.4 | 92.0 | 29.7 | 12.8 | $A_2A$ | 2.9 | 0.3 | Negative for $\alpha$-Thalassemia 2 and CS/PS gene |
| **10** | 4.99 | 14.9 | 44.5 | 89.1 | 29.8 | 12.1 | $A_2A$ | 2.4 | 0.2 | Negative for $\alpha$-Thalassemia 2 and CS/PS gene |

**Table S2.** Genotypic and Phenotypic Classification of Thalassemia of Normal Group: Alpha thalassemia 2 heterozygote (Group 22)

| No. | RBC | Hb (g/dl) | Hct (%) | MCV (fl) | MCH (pg) | RDW (%) | Hb Typing | HbA2/HbE (%) | HbF (%) | PCR for α-Thalassemia 2 3.7/4.2 kb deletion, CS/PS gene |
|---|---|---|---|---|---|---|---|---|---|---|
| **1** | 4.52 | 11.3 | 33.5 | 74.0 | 25.0 | 14.6 | $A_2A$ | 2.5 | - | Positive for α– thalassemia 2 gene (3.7 kb del) |
| **2** | 4.88 | 12.1 | 36.6 | 74.9 | 24.8 | 15.3 | $A_2A$ | 2.4 | 0.2 | Positive for α– thalassemia 2 gene (3.7 kb del) |
| **3** | 4.94 | 12.8 | 39.1 | 79.2 | 25.9 | 16.2 | $A_2A$ | 2.7 | 0.6 | Positive for α– thalassemia 2 gene (3.7 kb del) |
| **4** | 5.03 | 13.3 | 39.0 | 77.5 | 26.4 | 12.9 | $A_2A$ | 2.9 | 0.5 | Positive for α– thalassemia 2 gene (3.7 kb del) |
| **5** | 5.09 | 13.2 | 40.7 | 80.0 | 26.0 | 13.4 | $A_2A$ | 3.0 | 0.9 | Positive for α– thalassemia 2 gene (3.7 kb del) |
| **6** | 5.16 | 13.2 | 40.8 | 79.1 | 25.6 | 11.9 | $A_2A$ | 3.0 | 0.1 | Positive for α– thalassemia 2 gene (3.7 kb del) |
| **7** | 4.26 | 11.2 | 35.7 | 83.6 | 26.4 | 14.3 | $A_2A$ | 2.7 | 0.2 | Positive for α– thalassemia 2 gene (3.7 kb del) |
| **8** | 4.29 | 11.2 | 34.4 | 80.1 | 26.1 | 13.3 | $A_2A$ | 2.9 | 0.3 | Positive for α– thalassemia 2 gene (3.7 kb del) |
| **9** | 4.45 | 11.7 | 36.4 | 81.8 | 26.3 | 13.7 | $A_2A$ | 3.0 | 1.2 | Positive for α– thalassemia 2 gene (3.7 kb del) |
| **10** | 5.05 | 12.9 | 41.9 | 83.1 | 25.6 | 13.6 | $A_2A$ | 2.8 | 0.2 | Positive for α– thalassemia 2 gene (3.7 kb del) |

**Table S3.** Genotypic and Phenotypic Classification of Thalassemia of Normal Group: Others-1 (Group 25)

| No. | RBC | Hb (g/dl) | Hct (%) | MCV (fl) | MCH (pg) | RDW (%) | Hb Typing | HbA2/HbE (%) | HbF (%) | PCR for α-Thalassemia 2 3.7/4.2 kb deletion, CS/PS gene |
|---|---|---|---|---|---|---|---|---|---|---|
| **1** | 5.59 | 13.5 | 41 | 73.5 | 24.2 | 14.4 | $A_2A$ | 2.8 | 0.4 | Positive for Hb Paksé gene, Negative for α–thalassemia 2 gene |
| **2** | 6.29 | 16.2 | 49.4 | 78.5 | 25.8 | 14 | $A_2A$ | 2.8 | 0.4 | Positive for Hb Constant Spring gene, Negative for α–thalassemia 2 gene |
| **3** | 5.57 | 14.7 | 45.3 | 81 | 26.3 | 13.3 | $A_2A$ | 2.9 | 0.4 | Positive for Hb Constant Spring gene, Negative for α–thalassemia 2 gene |
| **4** | 5.06 | 13 | 40 | 79 | 26 | 13 | $A_2A$ | 2.9 | 0.4 | Positive for Hb Constant Spring gene, Negative for α–thalassemia 2 gene |
| **5** | 5.56 | 15 | 43 | 76.7 | 26.6 | 13.9 | $A_2A$ | 2.9 | 0.5 | Positive for Hb Constant Spring gene, Negative for α–thalassemia 2 gene |
| **6** | 4.52 | 11.9 | 37.1 | 82.2 | 26.3 | 15 | $A_2A$ | 3.4 | 2.1 | Positive for Hb Constant Spring gene, Negative for α–thalassemia 2 gene |
| **7** | 4.76 | 12.6 | 38.2 | 80.3 | 26.5 | 14 | $A_2A$ | 3.1 | 0.6 | Positive for Hb Constant Spring gene, Negative for α–thalassemia 2 gene |
| **8** | 4.3 | 11.3 | 35.2 | 81.7 | 26 | 13.1 | $A_2A$ | 2.7 | 0.5 | Positive for Hb Constant Spring gene, Negative for α–thalassemia 2 gene |
| **9** | 5.52 | 14.6 | 45.1 | 81.7 | 26.5 | 13.5 | $A_2A$ | 2.7 | 0.7 | Positive for Hb Constant Spring gene, Negative for α–thalassemia 2 gene |
| **10** | 5.12 | 13.4 | 40 | 78.1 | 26.2 | 12.8 | $A_2A$ | 2.8 | 0.7 | Positive for Hb Constant Spring gene, Negative for α–thalassemia 2 gene |

**Table S4.** Genotypic and Phenotypic Classification of Thalassemia of Carrier Group: Alpha (Group 21, 24 and 45)

| No. | RBC | Hb (g/dl) | Hct (%) | MCV (fl) | MCH (pg) | RDW (%) | Hb Typing | HbA2/HbE (%) | HbF (%) | Genotyping |
|---|---|---|---|---|---|---|---|---|---|---|
| **1** | 6.82 | 14.6 | 46.0 | 67.5 | 21.4 | - | 2.2 | | 0.4 | Alpha-thalassemia 1 heterozygote |
| **2** | 5.77 | 15.2 | 47.7 | 64.3 | 19.3 | 13.5 | 2.7 | 0.2 | 0.4 | Alpha-thalassemia 1 heterozygote |
| **3** | 4.78 | 8.4 | 28.5 | 59.6 | 17.6 | 24.8 | 2.5 | 0.3 | 0.4 | Alpha-thalassemia 1 heterozygote |
| **4** | 6.62 | 14.1 | 44.0 | 66.5 | 21.4 | 14.0 | 2.5 | 0.5 | 0.4 | Alpha-thalassemia 1 heterozygote |
| **5** | 6.07 | 12.0 | 39.7 | 65.4 | 19.8 | 16.0 | 2.3 | 0.0 | 0.5 | Alpha-thalassemia 1 heterozygote |
| **6** | 6.05 | 11.5 | 38.5 | 63.7 | 19.2 | 15.7 | 2.3 | 0.0 | 2.1 | Alpha-thalassemia 1 heterozygote |
| **7** | 6.94 | 14.2 | 46.0 | 66.9 | 20.5 | 15.2 | 2.2 | | 0.6 | Alpha-thalassemia 1 heterozygote |
| **8** | 6.73 | 14.7 | 43.0 | 64.0 | 21.8 | 17.5 | 2.8 | 0.7 | 0.5 | Alpha-thalassemia 1 heterozygote |
| **9** | 6.14 | 12.4 | 38.1 | 62.0 | 20.2 | 15.0 | 2.6 | 1.1 | 0.7 | Alpha-thalassemia 1 heterozygote |
| **10** | 5.97 | 12.4 | 38.3 | 64.2 | 20.7 | 15.2 | 2.9 | 0.6 | 0.7 | Alpha-thalassemia 1 heterozygote |
| **11** | 1549 | 5.72 | 13.5 | 43.1 | 75.3 | 23.6 | 12.5 | 3.2 | 0.2 | Compound Alpha-thalassemia 2 heterozygote |
| **12** | 5.67 | 15.2 | 46.7 | 83.9 | 27.8 | 14.9 | 25.7 | 11.1 | 5.67 | Hb E heterozygote with Alpha-thalassemia 2 heterozygote |

**Table S5.** Genotypic and Phenotypic Classification of Thalassemia of Carrier Group: Beta (Group 31 and 34)

| No. | RBC | Hb (g/dl) | Hct (%) | MCV (fl) | MCH (pg) | RDW (%) | Hb Typing | HbA2/HbE (%) | HbF (%) | Genotyping |
|---|---|---|---|---|---|---|---|---|---|---|
| **1** | 4.29 | 9.4 | 28.9 | 67.3 | 21.9 | 18.0 | 4.8 | 0.6 | 0.4 | Beta (0)-thalassemia heterozygote |
| **2** | 6.21 | 13.1 | 42.3 | 68.1 | 21.1 | 14.8 | 4.9 | 0.8 | 0.4 | Beta (0)-thalassemia heterozygote |
| **3** | 4.67 | 11.6 | 32.9 | 66.0 | 21.1 | 16.1 | 5.5 | 0.4 | 0.4 | Beta (0)-thalassemia heterozygote |
| **4** | 5.33 | 10.2 | 32.3 | 60.6 | 19.1 | 16.2 | 5.7 | 1.2 | 0.4 | Beta (0)-thalassemia heterozygote |
| **5** | 5.36 | 11.3 | 34.6 | 61.2 | 18.1 | 18.9 | 6.7 | 3.6 | 0.5 | Beta (0)-thalassemia heterozygote |
| **6** | 4.90 | 9.3 | 30.9 | 63.1 | 19.0 | 20.3 | 5.8 | 1.0 | 2.1 | Beta (0)-thalassemia heterozygote |
| **7** | 5.55 | 11.6 | 36.2 | 65.2 | 20.9 | 15.4 | 5.1 | 2.0 | 0.6 | Beta (0)-thalassemia heterozygote |
| **8** | 7.25 | 14.3 | 46.3 | 63.9 | 19.7 | 18.1 | 7.2 | 2.1 | 0.5 | Beta (0)-thalassemia heterozygote |
| **9** | 4.93 | 9.4 | 28.9 | 58.1 | 18.8 | 18.9 | 5.6 | 1.1 | 0.7 | Beta (0)-thalassemia heterozygote |
| **10** | 4.56 | 9.4 | 28.3 | 61.9 | 20.6 | 16.3 | 5.6 | 0.9 | 0.7 | Beta (0)-thalassemia heterozygote |

**Table S5. Continued** Genotypic and Phenotypic Classification of Thalassemia of Carrier Group: Beta (Group 31 and 34)

| No. | RBC | Hb (g/dl) | Hct (%) | MCV (fl) | MCH (pg) | RDW (%) | Hb Typing | HbA2/HbE (%) | HbF (%) | Genotyping |
|---|---|---|---|---|---|---|---|---|---|---|
| **1** | 4.65 | 10.3 | 33.0 | 71.0 | 22.1 | 15.7 | 5.6 | 1.2 | 4.65 | Beta (+)-thalassemia heterozygote |
| **2** | 4.66 | 10.8 | 33.5 | 72.0 | 23.3 | 18.4 | 4.7 | 1.1 | 4.66 | Beta (+)-thalassemia heterozygote |
| **3** | 5.75 | 12.0 | 37.7 | 65.7 | 65.6 | 20.9 | 5.7 | 2.2 | 5.75 | Beta (+)-thalassemia heterozygote |
| **4** | 6.21 | 14.0 | 42.3 | 68.0 | 22.5 | 16.6 | 6.0 | 0 | 6.21 | Beta (+)-thalassemia heterozygote |
| **5** | 6.21 | 14.0 | 42.3 | 68.0 | 22.5 | 16.6 | 6.0 | 0 | 6.21 | Beta (+)-thalassemia heterozygote |
| **6** | 5.90 | 13.6 | 42.0 | 71.1 | 32.1 | 15.0 | 6.0 | 1.1 | 5.90 | Beta (+)-thalassemia heterozygote |
| **7** | 4.48 | 11.3 | 34.4 | 76.9 | 25.2 | 18.0 | 4.8 | 0.6 | 4.48 | Beta (+)-thalassemia heterozygote |
| **8** | 5.81 | 12.6 | 39.7 | 68.4 | 21.6 | 15.5 | 5.6 | 1.9 | 5.81 | Beta (+)-thalassemia heterozygote |
| **9** | 5.64 | 12.9 | 38.7 | 68.6 | 22.8 | 16.1 | 6.2 | 2 | 5.64 | Beta (+)-thalassemia heterozygote |
| **10** | 4.88 | 10.6 | 33.2 | 68.1 | 21.7 | 15.3 | 5.6 | 1.3 | 4.88 | Beta (+)-thalassemia heterozygote |

**Table S6.** Genotypic and Phenotypic Classification of Thalassemia of Carrier Group: HbE Heterozygote (Group 32)

| No. | RBC | Hb (g/dl) | Hct (%) | MCV (fl) | MCH (pg) | RDW (%) | Hb Typing | HbA2/HbE (%) | HbF (%) | Genotyping |
|---|---|---|---|---|---|---|---|---|---|---|
| **1** | 4.33 | 11.3 | 34.3 | 75.4 | 24.8 | 19.1 | 28.4 | 0.4 | 4.33 | Hb E heterozygote |
| **2** | 5.06 | 11.8 | 35.0 | 67.2 | 23.3 | 14.6 | 29.2 | 1.5 | 5.06 | Hb E heterozygote |
| **3** | 3.63 | 9.7 | 28.6 | 78.0 | 26.6 | 14.9 | 29.6 | 0.8 | 3.63 | Hb E heterozygote |
| **4** | 3.36 | 8.8 | 26.5 | 78.9 | 26.1 | 17.2 | 29.4 | 0.7 | 3.36 | Hb E heterozygote |
| **5** | 4.60 | 11.0 | 32.0 | 70.0 | 23.9 | 15.6 | 28.1 | 1.7 | 4.60 | Hb E heterozygote |
| **6** | 2.49 | 6.8 | 20.9 | 83.9 | 27.3 | 15.9 | 31.5 | 0.4 | 2.49 | Hb E heterozygote |
| **7** | 5.43 | 13.8 | 41.5 | 77.7 | 25.8 | 14.6 | 27.4 | 0.7 | 5.43 | Hb E heterozygote |
| **8** | 4.86 | 9.7 | 29.8 | 65.4 | 21.3 | 16.6 | 28.0 | 0.8 | 4.86 | Hb E heterozygote |
| **9** | 3.81 | 10.0 | 29.6 | 77.6 | 26.4 | 14.0 | 25.1 | 0.7 | 3.81 | Hb E heterozygote |
| **10** | 5.57 | 13.5 | 39.8 | 72.0 | 24.0 | 16.0 | 25.2 | 1.0 | 5.57 | Hb E heterozygote |

**Table S7.** Genotypic and Phenotypic Classification of Thalassemia of Carrier Group: Hb E heterozygote with Alpha-thalassemia 1 heterozygote (Group 44)

| No. | RBC | Hb (g/dl) | Hct (%) | MCV (fl) | MCH (pg) | RDW (%) | Hb Typing | HbA2/HbE (%) | HbF (%) | Genotyping |
|---|---|---|---|---|---|---|---|---|---|---|
| **1** | 6.32 | 12.7 | 39.7 | 62.8 | 20.1 | 17.2 | 18.7 | 0.5 | 6.32 | Hb E heterozygote with Alpha-thalassemia 1 heterozygote |
| **2** | 5.65 | 11.8 | 36.9 | 65.3 | 20.9 | 17.2 | 18.9 | 0.8 | 5.65 | Hb E heterozygote with Alpha-thalassemia 1 heterozygote |
| **3** | 6.11 | 13.3 | 42.8 | 70.0 | 21.8 | 14.7 | 19.1 | 0.5 | 6.11 | Hb E heterozygote with Alpha-thalassemia 1 heterozygote |
| **4** | 5.70 | 11.6 | 36.0 | 63.0 | 20.0 | 16.0 | 20.9 | 1.2 | 5.70 | Hb E heterozygote with Alpha-thalassemia 1 heterozygote |
| **5** | 6.32 | 14.1 | 40.7 | 64.0 | 22.0 | 17.0 | 19.1 | 0.6 | 6.32 | Hb E heterozygote with Alpha-thalassemia 1 heterozygote |
| **6** | 6.32 | 14.1 | 40.7 | 64.0 | 22.0 | 17.0 | 19.1 | 0.6 | 6.32 | Hb E heterozygote with Alpha-thalassemia 1 heterozygote |
| **7** | 5.33 | 10.6 | 33.5 | 62.9 | 19.9 | 15.3 | 18.6 | 1.0 | 5.33 | Hb E heterozygote with Alpha-thalassemia 1 heterozygote |
| **8** | 5.62 | 10.9 | 35.4 | 63.0 | 19.3 | 18.2 | 20.0 | 0.4 | 5.62 | Hb E heterozygote with Alpha-thalassemia 1 heterozygote |
| **9** | 5.30 | 11.7 | 36.4 | 68.6 | 22.1 | 13.4 | 20.2 | 0.6 | 5.30 | Hb E heterozygote with Alpha-thalassemia 1 heterozygote |
| **10** | 5.43 | 11.5 | 37.0 | 68.1 | 21.2 | 15.3 | 19.4 | 0.4 | 5.43 | Hb E heterozygote with Alpha-thalassemia 1 heterozygote |

**Table S8.** Genotypic and Phenotypic Classification of Thalassemia of Carrier Group: Others-2 (Group 39)

| No. | RBC | Hb (g/dl) | Hct (%) | MCV (fl) | MCH (pg) | RDW (%) | Hb Typing | HbA2/HbE (%) | HbF (%) | Genotyping |
|---|---|---|---|---|---|---|---|---|---|---|
| **1** | 5.50 | 12.5 | 38.0 | 68.7 | 22.7 | 15.9 | 28.1 | 1.2 | 5.50 | Hb E heterozygote with Alpha-thalassemia 2 heterozygote? |
| **2** | 6.63 | 18.5 | 56.0 | 84.8 | 27.9 | 12.1 | 22.3 | 0.9 | 6.63 | Hb E heterozygote with Alpha-thalassemia 2 heterozygote? |
| **3** | 5.02 | 12.6 | 36.8 | 73.3 | 25.1 | 15.5 | 23.2 | 0.8 | 5.02 | Hb E heterozygote with Alpha-thalassemia 2 heterozygote? |
| **4** | 6.11 | 15.2 | 47.1 | 77.1 | 24.8 | 14.8 | 24.3 | 0.7 | 6.11 | Hb E heterozygote with Alpha-thalassemia 2 heterozygote? |
| **5** | 4.92 | 11.2 | 35.1 | 71.2 | 22.8 | 14.6 | 21.8 | 0.9 | 4.92 | Hb E heterozygote with Alpha-thalassemia 2 heterozygote? |
| **6** | 6.26 | 15.2 | 48.9 | 78.1 | 24.3 | 14.8 | 23.8 | 0.6 | 6.26 | Hb E heterozygote with Alpha-thalassemia 2 heterozygote? |
| **7** | 5.10 | 13.9 | 42.0 | 81.7 | 27.3 | 13.6 | 24.7 | 0.5 | 5.10 | Hb E heterozygote with Alpha-thalassemia 2 heterozygote? |
| **8** | 4.74 | 10.4 | 32.0 | 66.9 | 22.0 | 18.6 | 18.3 | 1.9 | 4.74 | Hb E heterozygote with Alpha-thalassemia 2 heterozygote? |
| **9** | 6.37 | 15.9 | 48.2 | 75.7 | 25.0 | 13.7 | 23.6 | 0.6 | 6.37 | Hb E heterozygote with Alpha-thalassemia 2 heterozygote? |
| **10** | 6.32 | 14.6 | 43.0 | 67.6 | 23.1 | 15.4 | 20.1 | 1.1 | 6.32 | Hb E heterozygote with Alpha-thalassemia 2 heterozygote? |

**Table S9.** Genotypic and Phenotypic Classification of Thalassemia of Disease+Symptom Group: Hb E homozygote (Group 33)

| No. | RBC | Hb (g/dl) | Hct (%) | MCV (fl) | MCH (pg) | RDW (%) | Hb Typing | HbA2/HbE (%) | HbF (%) | Genotyping |
|---|---|---|---|---|---|---|---|---|---|---|
| **1** | 4.69 | 11.8 | 39.1 | 60.3 | 18.2 | 16.6 | 96.0 | 1.8 | 4.69 | Hb E homozygote |
| **2** | 6.38 | 12.8 | 37.5 | 59.0 | 20.1 | 17.7 | 95.6 | 2.5 | 6.38 | Hb E homozygote |
| **3** | 5.75 | 11.1 | 33.0 | 56.7 | 19.3 | 16.5 | 82.3 | 4.0 | 5.75 | Hb E homozygote |
| **4** | 4.48 | 9.3 | 27.9 | 62.3 | 20.7 | 16.8 | 82.9 | 4.6 | 4.48 | Hb E homozygote |
| **5** | 6.13 | 12.2 | 35.5 | 57.9 | 19.9 | 16.0 | 80.8 | 2.4 | 6.13 | Hb E homozygote |
| **6** | 4.55 | 9.2 | 27.5 | 60.0 | 20.2 | 17.0 | 78.3 | 3.6 | 4.55 | Hb E homozygote |
| **7** | 5.39 | 10.9 | 33.8 | 62.7 | 20.2 | 16.6 | 93.5 | 4.2 | 5.39 | Hb E homozygote |
| **8** | 6.00 | 12.5 | 38.0 | 63.3 | 20.8 | 16.2 | 81.4 | 0.9 | 6.00 | Hb E homozygote |
| **9** | 5.36 | 10.9 | 32.4 | 60.4 | 20.3 | 17.1 | 87.0 | 4.3 | 5.36 | Hb E homozygote |
| **10** | 5.94 | 12.0 | 37.5 | 63.1 | 20.2 | 16.6 | 87.8 | 2.6 | 5.94 | Hb E homozygote |

**Table S10.** Genotypic and Phenotypic Classification of Thalassemia of Disease+Symptom Group: Hb E homozygote with Alpha-thalassemia 1 heterozygote (Group 47)

| No. | RBC | Hb (g/dl) | Hct (%) | MCV (fl) | MCH (pg) | RDW (%) | Hb Typing | HbA2/HbE (%) | HbF (%) | Genotyping |
|---|---|---|---|---|---|---|---|---|---|---|
| **1** | 4.56 | 10.3 | 28.4 | 62.0 | 22.6 | 18.5 | 81.7 | 9.7 | 4.56 | Hb E homozygote with Alpha-thalassemia 1 heterozygote |
| **2** | 5.54 | 11.2 | 32.8 | 59.0 | 20.2 | 18.3 | 87.8 | 3.6 | 5.54 | Hb E homozygote with Alpha-thalassemia 1 heterozygote |
| **3** | 7.07 | 11.3 | 33.0 | 46.5 | 15.9 | 17.4 | 97.4 | 2.6 | 7.07 | Hb E homozygote with Alpha-thalassemia 1 heterozygote |
| **4** | 6.77 | 13.4 | 42.0 | 61.7 | 19.8 | 18.0 | 81.8 | 3.7 | 6.77 | Hb E homozygote with Alpha-thalassemia 1 heterozygote |
| **5** | 7.41 | 13.2 | 42.4 | 57.2 | 17.9 | 17.9 | 85.9 | 1.6 | 7.41 | Hb E homozygote with Alpha-thalassemia 1 heterozygote |
| **6** | 5.74 | 11.4 | 36.0 | 62.7 | 19.9 | 16.4 | 78.2 | 5.0 | 5.74 | Hb E homozygote with Alpha-thalassemia 1 heterozygote |
| **7** | 6.37 | 13.3 | 41.2 | 64.8 | 20.9 | 15.3 | 91.0 | 7.0 | 6.37 | Hb E homozygote with Alpha-thalassemia 1 heterozygote |

**Table S11.** Genotypic and Phenotypic Classification of Thalassemia of Disease+Symptom Group: Hb H disease (Group 51)

| No. | RBC | Hb (g/dl) | Hct (%) | MCV (fl) | MCH (pg) | RDW (%) | Hb Typing | HbA2/HbE (%) | HbF (%) | Genotyping |
|---|---|---|---|---|---|---|---|---|---|---|
| **1** | 5.19 | 9.0 | 46.8 | 56.3 | 18.2 | 24.7 | 2.3 | 0.5 | 5.19 | Hb H disease |
| **2** | 5.26 | 9.3 | 49.2 | 56.3 | 18.0 | 24.5 | 2.2 | 0.5 | 5.26 | Hb H disease |
| **3** | 5.15 | 9.1 | 49.2 | 59.8 | 18.1 | 23.8 | 2.0 | 0.5 | 5.15 | Hb H disease |
| **4** | 4.92 | 8.8 | 30.9 | 60.0 | 18.0 | 23.9 | 2.0 | 0.4 | 4.92 | Hb H disease |
| **5** | 5.02 | 9.0 | 31.7 | 64.5 | 18.2 | 24.6 | 2.0 | 0.4 | 5.02 | Hb H disease |
| **6** | 5.18 | 9.2 | 32.0 | 63.3 | 18.1 | 24.9 | 3.1 | 0.4 | 5.18 | Hb H disease |
| **7** | 5.41 | 9.6 | 33.2 | 62.8 | 18.3 | 24.7 | 3.2 | 0.5 | 5.41 | Hb H disease |
| **8** | 4.95 | 8.8 | 62.2 | 65.1 | 18.8 | 25.2 | 3.5 | 0.5 | 4.95 | Hb H disease |
| **9** | 4.64 | 8.5 | 61.1 | 66.2 | 19.0 | 24.4 | 3.5 | 0.5 | 4.64 | Hb H disease |
| **10** | 3.87 | 7.7 | 24.5 | 85.5 | 27.5 | 20.6 | 2.7 | 0.3 | 3.87 | Hb H disease |

**Table S12.** Genotypic and Phenotypic Classification of Thalassemia of Disease+Symptom Group: Others-3 (Group 89)

| No. | RBC | Hb (g/dl) | Hct (%) | MCV (fl) | MCH (pg) | RDW (%) | Hb Typing | HbA2/HbE (%) | HbF (%) | Genotyping |
|---|---|---|---|---|---|---|---|---|---|---|
| **1** | 5.97 | 11.6 | 36.8 | 60.1 | 18.9 | 17.8 | 16.7 | 3.5 | 5.97 | EA Bart's diseases? |
| **2** | 6.04 | 11.9 | 37.3 | 59.9 | 19.0 | 17.8 | 14.7 | 1.6 | 6.04 | EA Bart's diseases? |
| **3** | 5.92 | 11.5 | 36.0 | 59.5 | 18.9 | 17.6 | 15.6 | 3.5 | 5.92 | EA Bart's diseases? |
| **4** | 5.62 | 11.3 | 34.7 | 61.7 | 20.1 | 16.6 | 16.7 | 0.7 | 5.62 | EA Bart's diseases? |
| **5** | 6.61 | 15.0 | 46.8 | 70.7 | 22.6 | 16.0 | 16.0 | 3.9 | 6.61 | EA Bart's diseases? |
| **6** | 7.13 | 15.3 | 47.1 | 66.0 | 21.4 | 15.4 | 17.2 | 3.9 | 7.13 | EA Bart's diseases? |
| **7** | 4.69 | 7.3 | 22.3 | 47.6 | 15.7 | 24.6 | 15.6 | 0.4 | 4.69 | EA Bart's diseases? |
| **8** | 5.57 | 8.7 | 28.5 | 51.1 | 15.5 | 16.7 | 14.7 | 1.0 | 5.57 | EA Bart's diseases? |
| **9** | 5.00 | 10.4 | 30.9 | 61.7 | 20.7 | 19.1 | 17.1 | 0.9 | 5.00 | EA Bart's diseases? |
| **10** | 4.46 | 7.8 | 23.6 | 52.8 | 17.5 | 19.6 | 14.1 | 1.6 | 4.46 | EA Bart's diseases? |
